# Supplementary material for: Diversity and potential host-interactions of viruses inhabiting deep-sea seamount sediments
Source: Nat Commun. 2024 Apr 15;15:3228. doi: 10.1038/s41467-024-47600-1 (PMC11018836; doi:10.1038/s41467-024-47600-1)
Supplement: Supplementary file 1 — Supplementary Information [file 41467_2024_47600_MOESM1_ESM.pdf]

Supplementary Information

Diversity and potential host-interactions of viruses inhabiting deep-sea seamount sediments

Meishun Yu[1†], Menghui Zhang[1†], Runying Zeng[1], Ruolin Cheng[1], Rui Zhang[2], Yanping Hou[1], Fangfang Kuang[1], Xuejin Feng[1], Xiyang Dong[1], Yinfang Li[1], Zongze Shao[1*], Min Jin[1*]

[1]State Key Laboratory Breeding Base of Marine Genetic Resource and Southern Marine Science and Engineering Guangdong Laboratory (Zhuhai), Third Institute of Oceanography, Ministry of Natural Resources, Xiamen, 361000, China

[2]Institute for Advanced Study, Shenzhen University, Shenzhen, Guangdong, China

† These authors contributed equally to this work.

*Corresponding author (Min Jin):

E-mail address: jinmin@tio.org.cn

*Corresponding author (Zongze Shao):

E-mail address: shaozongze@tio.org.cn

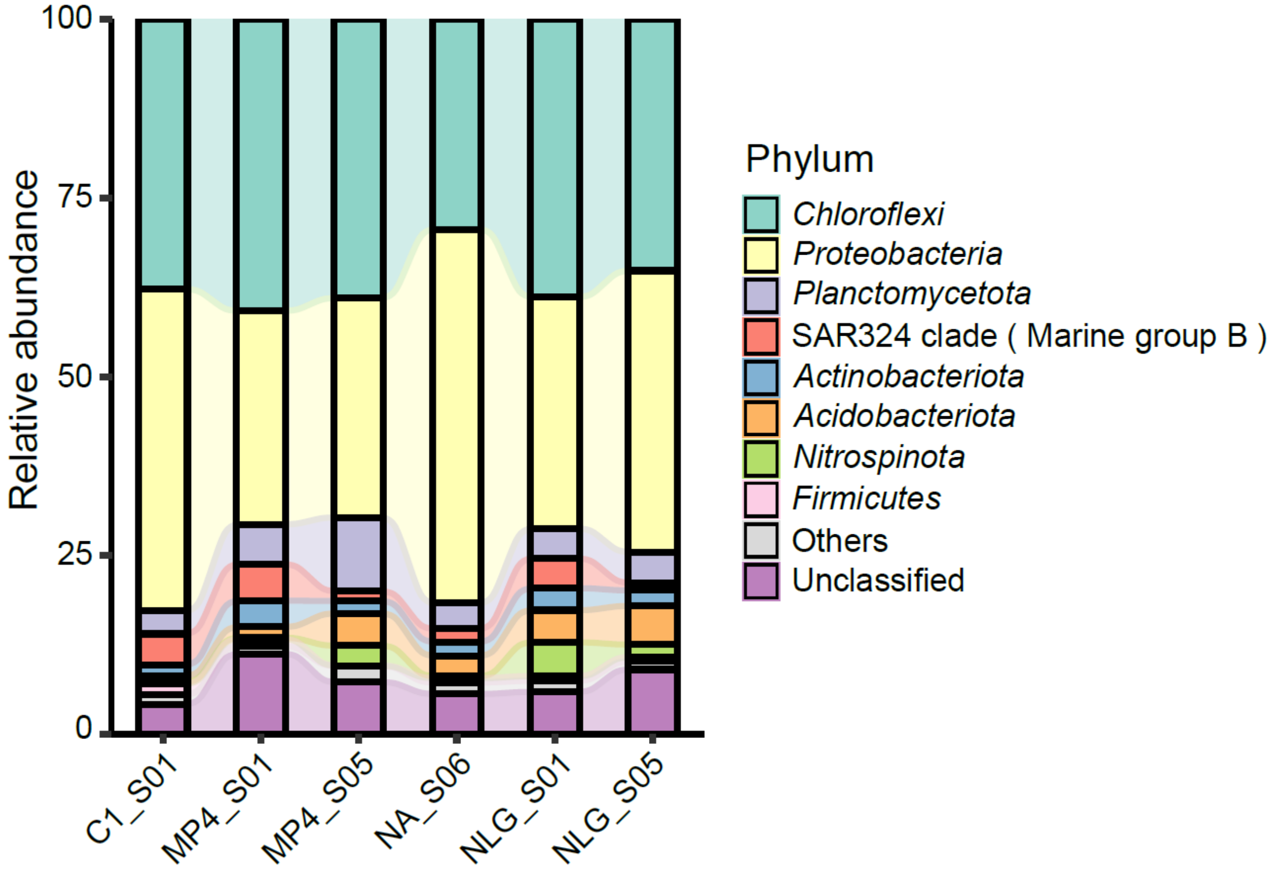

Supplementary Fig. 1 Relative abundances of dominant prokaryotic communities in seamount sediments at the phylum level.

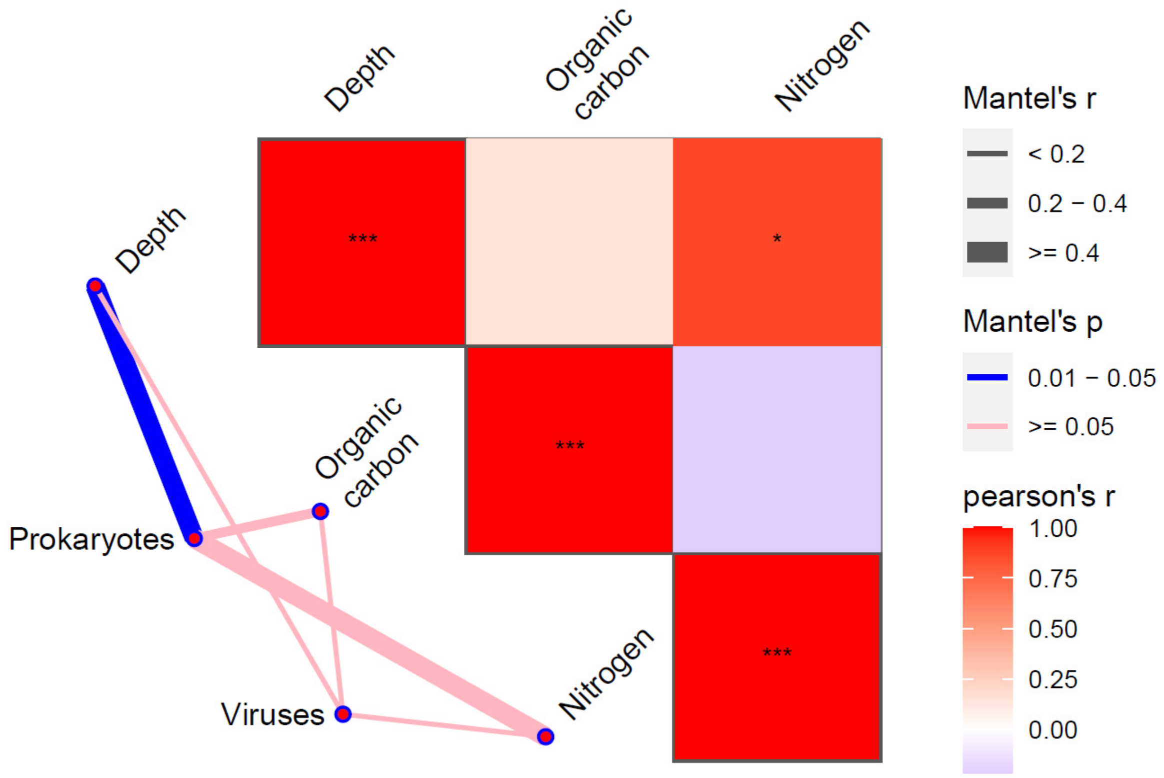

Supplementary Fig. 2 Mantel's correlation analysis between seamount sediment environmental variables and prokaryotic and viral communities (n = 6 samples). Prokaryotic and viral operational taxonomic units (OTUs) profiles were related to depth, organic carbon, and nitrogen by the Mantel test. The degree of correlation is indicated by the thickness of the lines, and correlations with statistical significance ($p < 0.05$) are colored in blue. Pairwise comparisons of environmental variables are indicated with a color gradient denoting Pearson's correlation coefficient (*$p < 0.05$, **$p < 0.01$, ***$p < 0.001$). The exact *p* values are indicated in the Source Data file.

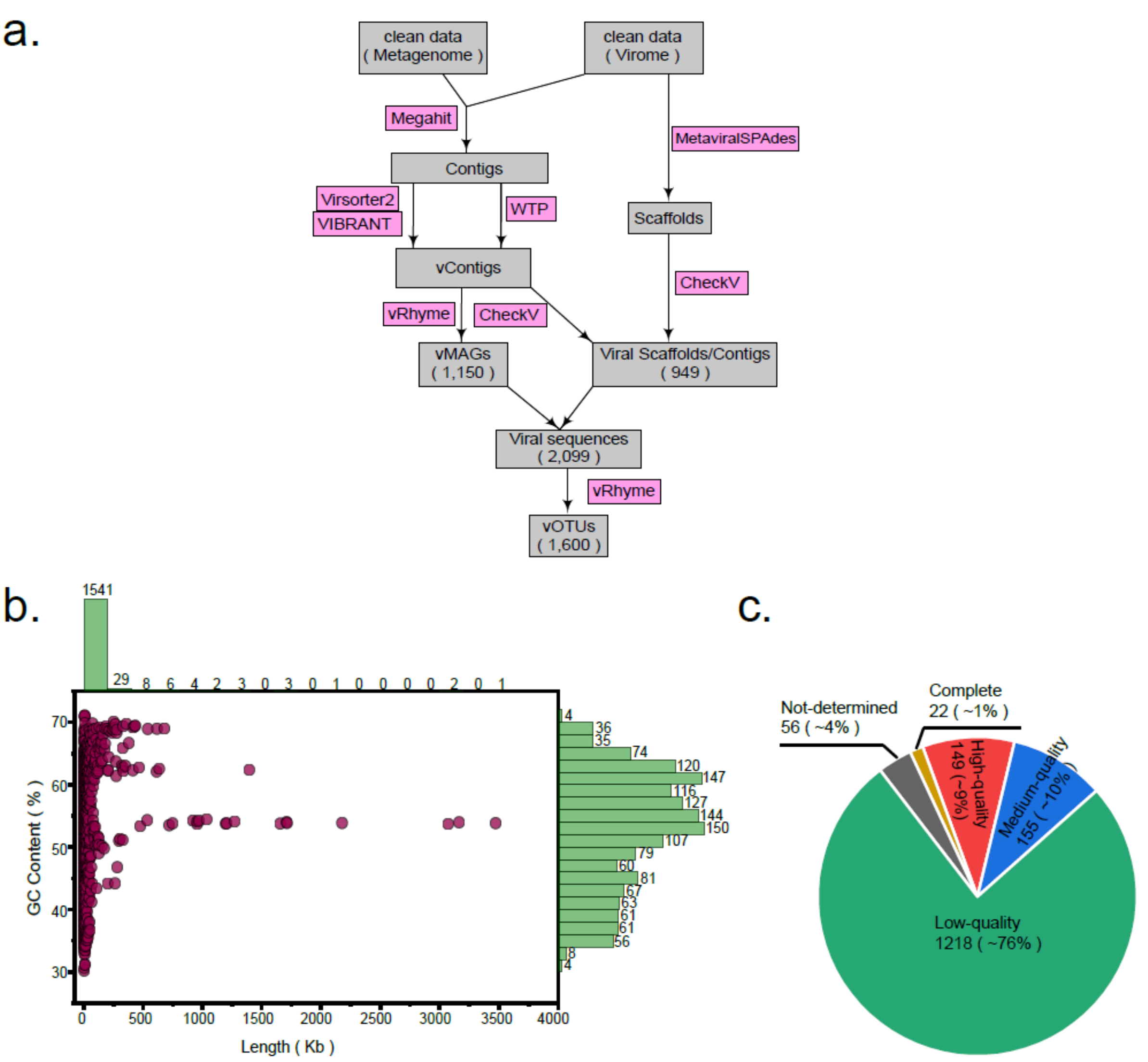

Supplementary Fig. 3 Identification and characteristics of viral operational taxonomic units (vOTUs). (a) An overview of the bioinformatic workflow used to identify vOTUs. Pink and grey boxes indicate the software used and output results, respectively. (b) Distribution of 1600 vOTUs by length and GC content. The green bar charts indicate the numbers of vOTUs in each category. (c) Distribution of 1600 vOTUs, quality assessed by CheckV.

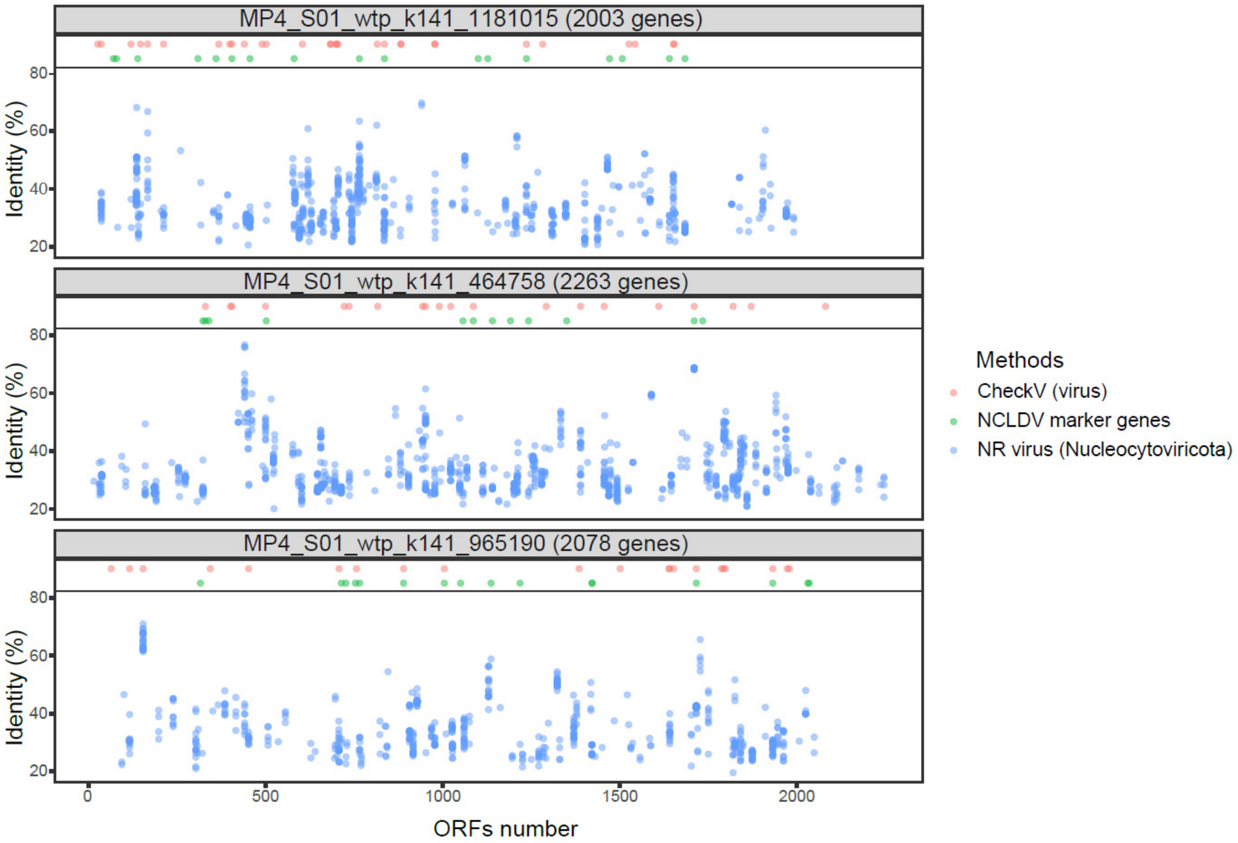

Supplementary Fig. 4 The open reading frame (ORF) annotation of three largest putative nucleocytoplasmic large DNA virus (NCLDV) genomes. The ORFs identified as viral genes by CheckV v0.9.0[1] are marked with red dots, while the ORFs annotated as Nucleocytoviricota marker genes by ncldv_markersearch[2] are marked with green dots. The ORFs were searched against the Nucleocytoviricota genomes of the NR_virus database, and their homology is indicated by blue dots.

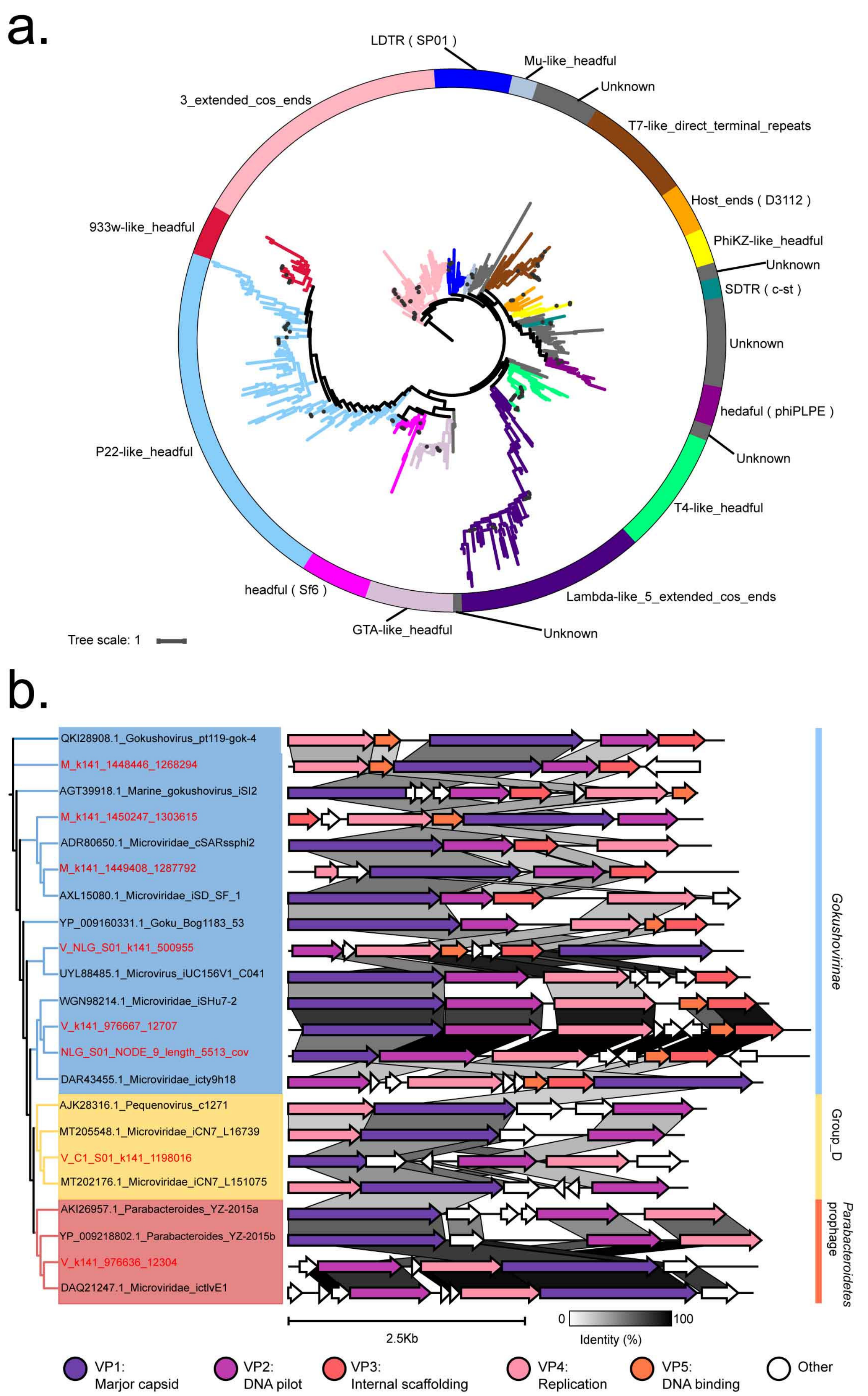

Supplementary Fig. 5 Phylogenetic trees of major virus groups in seamount sediments. (a) Maximum-likelihood phylogenetic tree of *Caudovirales* based on terminase large subunit (TerL). The reference sequences (Supplementary Data 4) are marked with black dots, and phage packaging mechanisms are marked with different colours in the

outer ring. (b) VP1 phylogeny and genome structure of *Microviridae.* The phylogenetic tree was inferred based on the Maximum-likelihood method. The reference sequences (Supplementary Data 13) and seamount viral sequences are marked in red and black in the tree, respectively. The genome structures of microviruses recovered from seamount sediment were compared to other *Microviridae* genomes (see Supplementary Data 13), and the level of similarity is indicated by grey shading. Homologous genes are colour-coded according to the legend.

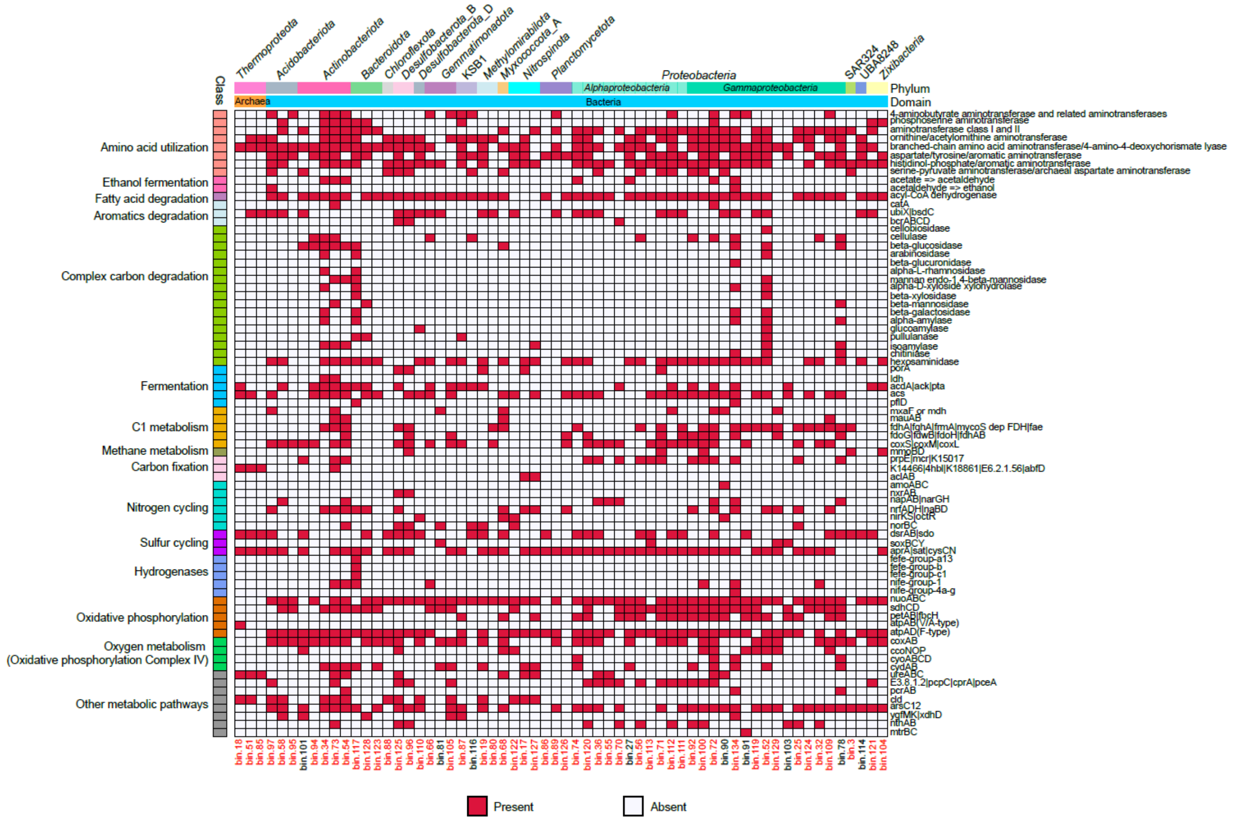

Supplementary Fig. 6 Heatmap showing the presence (red) and absence (white) of metabolic genes in metagenome-assembled genomes (MAGs). The taxonomic annotation of MAGs is indicated on the top, and the function class for each metabolic gene is indicated on the left. The MAGs that were linked with vOTUs are marked in red.

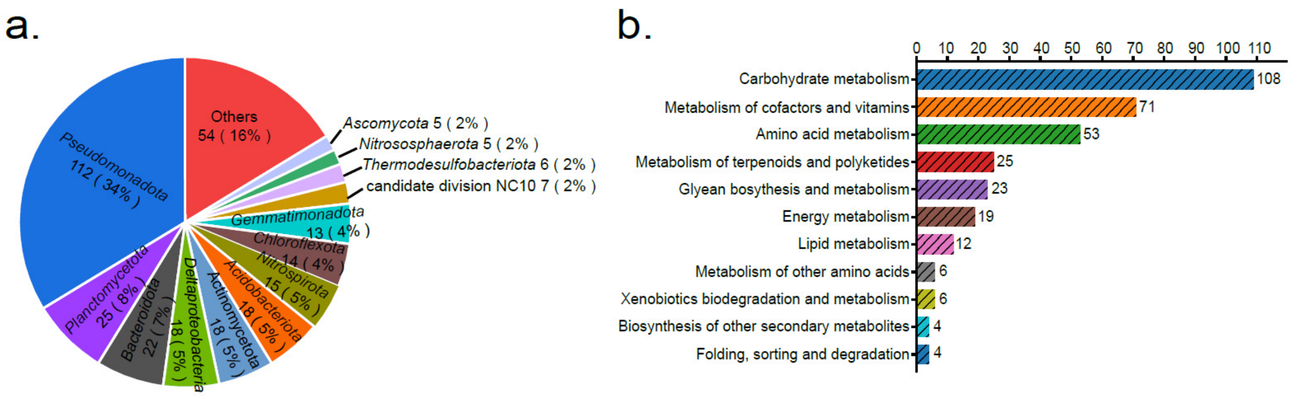

Supplementary Fig. 7 Functional classification and origins of viral auxiliary metabolic genes (AMGs). (a) Predicted hosts of viral AMGs. (b) Kyoto Encyclopedia of Genes and Genomes (KEGG) annotation of viral AMGs.

References

1. Nayfach, S., *et al.* CheckV assesses the quality and completeness of metagenome-assembled viral genomes. *Nat. Biotechnol.* **39**, 578-585 (2021).

2. Moniruzzaman, M., Martinez-Gutierrez, C. A., Weinheimer, A. R., & Aylward, F. O. Dynamic genome evolution and complex virocell metabolism of globally-distributed giant viruses. *Nat. commun.* **11**, 1710 (2020).
