# Supplementary material for: Diversity and potential host-interactions of viruses inhabiting deep-sea seamount sediments
Source: Nat Commun. 2024 Apr 15;15:3228. doi: 10.1038/s41467-024-47600-1 (PMC11018836; doi:10.1038/s41467-024-47600-1)
Supplement: Supplementary file 3 — Description of Additional Supplementary Files [file 41467_2024_47600_MOESM3_ESM.docx]

File Name: Supplementary Data 1
Description: Descriptions and general high-throughput sequencing features of seamount samples

File Name: Supplementary Data 2
Description: Characteristics of 62 seamount prokaryotic metagenome-assembled genomes (MAGs)

File Name: Supplementary Data 3
Description: Characteristics of 1600 vOTUs and their relative abundance in each sample

File Name: Supplementary Data 4
Description: Reference sequences of terminase large subunits (terL) with different packaging mechanisms

File Name: Supplementary Data 5
Description: Clustered vOTUs among seamount sediments, cold seep, trench, seawater and viral RefSeq database based on vConTACT2. One-sided Mann–Whitney U-test was used to assess the quality of clustering

File Name: Supplementary Data 6
Description: Occurrence of viral clusters among seamount sediments, cold seep sediments, trench, seawater and viral RefSeq database based on vConTACT2

File Name: Supplementary Data 7
Description: List of seamount MAGs obtained from IMG/M database

File Name: Supplementary Data 8
Description: List of putative virus-host linkages

File Name: Supplementary Data 9
Description: Metabolic capabilities of host genomes that predicted to be infected by viruses

File Name: Supplementary Data 10
Description: List of putative viral auxiliary metabolic genes

File Name: Supplementary Data 11
Description: Genomic context of selected viral AMGs

File Name: Supplementary Data 12
Description: Linkage of AMG-carrying viruses to the hosts

File Name: Supplementary Data 13
Description: Reference sequences of VP1 proteins from different *Microviridae* subfamilies
