# Supplementary material for: Diversity and potential host-interactions of viruses inhabiting deep-sea seamount sediments
Source: Nat Commun. 2024 Apr 15;15:3228. doi: 10.1038/s41467-024-47600-1 (PMC11018836; doi:10.1038/s41467-024-47600-1)
Supplement: Supplementary file 5 — Reporting Summary [file 41467_2024_47600_MOESM5_ESM.pdf]

nature portfolio

Corresponding author(s): Min Jin

Last updated by author(s): Feb 20, 2024

Reporting Summary

Nature Portfolio wishes to improve the reproducibility of the work that we publish. This form provides structure for consistency and transparency in reporting. For further information on Nature Portfolio policies, see our Editorial Policies and the Editorial Policy Checklist.

Statistics

For all statistical analyses, confirm that the following items are present in the figure legend, table legend, main text, or Methods section.

n/a	Confirmed	
☐	☒	The exact sample size (*n*) for each experimental group/condition, given as a discrete number and unit of measurement
☒	☐	A statement on whether measurements were taken from distinct samples or whether the same sample was measured repeatedly
☐	☒	The statistical test(s) used AND whether they are one- or two-sided<br>*Only common tests should be described solely by name; describe more complex techniques in the Methods section.*
☒	☐	A description of all covariates tested
☒	☐	A description of any assumptions or corrections, such as tests of normality and adjustment for multiple comparisons
☒	☐	A full description of the statistical parameters including central tendency (e.g. means) or other basic estimates (e.g. regression coefficient) AND variation (e.g. standard deviation) or associated estimates of uncertainty (e.g. confidence intervals)
☐	☒	For null hypothesis testing, the test statistic (e.g. *F*, *t*, *r*) with confidence intervals, effect sizes, degrees of freedom and *P* value noted<br>*Give P values as exact values whenever suitable.*
☒	☐	For Bayesian analysis, information on the choice of priors and Markov chain Monte Carlo settings
☒	☐	For hierarchical and complex designs, identification of the appropriate level for tests and full reporting of outcomes
☐	☒	Estimates of effect sizes (e.g. Cohen's *d*, Pearson's *r*), indicating how they were calculated

Our web collection on statistics for biologists contains articles on many of the points above.

Software and code

Policy information about availability of computer code

Data collection	No software was used for data collection
Data analysis	Metagenomic raw reads were trimmed and quality filtered using fastp v0.23.2. Contigs were assembled using MEGAHIT v1.2.9, quality assessed using QUAST v5.2.0, and binned by the MetaWRAP v1.3.0. The original bins were refined using the MetaWRAP v1.3.0, and quality checked by CheckM v1.0.12. The high- and medium-quality bins were aggregated and dereplicated using dRrep v3.3.0. MAGs were taxonomically assigned using GTDB-Tk v2.1.0. Maximum-likelihood phylogeny of MAGs was inferred using IQ-TREE v2.2.0.3 from a concatenation of marker genes produced by GTDB-Tk v2.1.0. Phylogeny tree was visualised using iTOL v4. Clean reads were mapped to MAGs using CoverM v0.6.1. Functional annotation of MAGs was performed using METABOLIC v4.0.<br>Virome raw reads were trimmed and quality filtered using fastp v0.23.2. Contigs were assembled using MEGAHIT v1.2.9. Viral sequences were identified by the pipelines of VirSorter2 v2.2.3, VIBRANT v1.2.0, What the Phage (Wtp) v1.1.0, and Metaviral SPAdes v3.15.5. Viral sequences were checked by CheckV v0.9.0 and binned using vRhyme v1.1.0. ORFs were predicted by Prodigal v2.6.3. Taxonomic affiliation of vOTUs was performed by CAT v5.0.3. The relative abundance of vOTUs was determined by CoverM v0.6.1. CRISPR spacer database was constructed using MinCED v0.4.2. CRISPR spacers were then queried using BLAST+ v2.9.0. tRNAs were identified using ARAGORN v1.2.41, and matched using BLASTn v2.9.0. Sequences of vOTUs were compared with the dataset of microbial genomes by BLASTn v2.9.0. WIsH v1.0 was run to infer a connection between viruses and hosts based on k-mer frequencies. Life strategies of vOTUs were predicted by the pipelines of VIBRANT v1.2.0 and CheckV v0.9.0. VP1 and TerL sequences were recovered using HMMER v3.3.2, and were aligned by MUSCLE v5.1. The alignments were trimmed using TrimAL v1.4.rev15. The phylogenetic tree was constructed using IQ-TREE v2.2.0.3, and visualised by iTOL v4. Viral AMGs were identified and annotated using both VIBRANT v1.2.0 and DRAMv v1.3.5 pipelines. Protein-sharing network analysis was conducted using vConTACT2 v0.11.3, and visualised using Cytoscape v3.9.1. ORFs were called using Prodigal v2.6.3 and were aligned to database using Diamond v2.0.15. ORFs were translated into proteins and clustered using CD-HIT v4.6 to generate PCs. Box plots, heat maps, bar stacking plots, and gene maps were drawn using the R packages ggplot2 v4.3.2, pheatmap v1.0.12, ggpubr v0.6.0, and gggenes v0.5.0,

respectively. Venn and upset plots were plotted by Tbtools v1.120. The R v4.1.0 package vegan v2.6-4 was used to calculate the Bray-Curtis distance matrix. The Pearson correlations and the Mantel test were performed and visualized using R software via the "ggcor" package.

For manuscripts utilizing custom algorithms or software that are central to the research but not yet described in published literature, software must be made available to editors and reviewers. We strongly encourage code deposition in a community repository (e.g. GitHub). See the Nature Portfolio guidelines for submitting code & software for further information.

Data

Policy information about availability of data

All manuscripts must include a data availability statement. This statement should provide the following information, where applicable:

- Accession codes, unique identifiers, or web links for publicly available datasets
- A description of any restrictions on data availability
- For clinical datasets or third party data, please ensure that the statement adheres to our policy

The raw data of bulk metagenome, virome, and 16S rRNA genes generated in this study have been deposited in the NCBI BioProject databases under accession code 1005434 [https://www.ncbi.nlm.nih.gov/bioproject/?term=PRJNA1005434]. All processed data generated in this study are provided in Supplementary Tables (Supplementary Table 1-13). Source data for all main and supplementary figures are provided with this paper. The Hidden Markov Models used in this study are available in the Pfam database under accession code PF03237 [https://www.ebi.ac.uk/interpro/entry/pfam/PF03237/], PF04466 [https://www.ebi.ac.uk/interpro/entry/pfam/PF04466/], PF05876 [https://www.ebi.ac.uk/interpro/entry/pfam/PF05876/], and PF02305 [https://www.ebi.ac.uk/interpro/entry/pfam/ PF02305/]. The links to the databases used in this study are listed below: Silva database (release 132) [https://www.arb-silva.de/documentation/release-132/]; Genome Taxonomy database [https://data.ace.uq.edu.au/public/gtdb/data/releases/release207/]; NCBI RefSeq database [https://ftp.ncbi.nlm.nih.gov/refseq/release/viral]/; NCBI Taxonomy database [https://www.ncbi.nlm.nih.gov/taxonomy]; eggNOG database (release 5.0) [http://eggnog5.embl.de/download/eggnog_5.0/]; Pfam [https://pfam.xfam.org/]; dbCAN2 server [https://bcb.unl.edu/dbCAN2/]; NCBI CD-search tool [https://www.ncbi.nlm.nih.gov/Structure/cdd/wrpsb.cgi]; Global Oceans Viromes 2 (GOV 2.0) database [https://datacommons.cyverse.org/browse/iplant/home/shared/iVirus/GOV2.0]; viral Contigs from cold seep [https://doi.org/10.6084/m9.figshare.12922229]; viral Contigs from trench (OEP001086 and OEP001087) [https://www.biosino.org/node/]; IMG/VR database (release 2022-12-19_7.1) [https://genome.jgi.doe.gov/portal/IMG_VR/IMG_VR.home.html]; IMG/MR [https://img.jgi.doe.gov/].

Research involving human participants, their data, or biological material

Policy information about studies with human participants or human data. See also policy information about sex, gender (identity/presentation), and sexual orientation and race, ethnicity and racism.

Reporting on sex and gender	not applicable
Reporting on race, ethnicity, or other socially relevant groupings	not applicable
Population characteristics	not applicable
Recruitment	not applicable
Ethics oversight	not applicable

Note that full information on the approval of the study protocol must also be provided in the manuscript.

Field-specific reporting

Please select the one below that is the best fit for your research. If you are not sure, read the appropriate sections before making your selection.

☐ Life sciences ☐ Behavioural & social sciences ☒ Ecological, evolutionary & environmental sciences

For a reference copy of the document with all sections, see nature.com/documents/nr-reporting-summary-flat.pdf

Ecological, evolutionary & environmental sciences study design

All studies must disclose on these points even when the disclosure is negative.

Study description	To gain insight into the community structure, genetic diversity, and ecological roles of viruses in seamount ecosystems, as well as how seamount geographic features influence local viral communities, we utilised a combination of 16S rRNA gene sequences, metagenome, and virome to study prokaryotic and viral communities in sediment samples collected from three seamounts in the western Pacific Ocean
Research sample	Seven deep-sea seamount sediment samples were collected from three adjacent seamounts in the Northwest Pacific. We chose them because they are sampled on a mesoscale level, which allows us to investigate the effects of the geographic features of seamounts on the microdiversity and biogeography of viruses. To fully consider the effect of the geographic features of seamounts on viral communities, sediment samples were collected across the seamount region, encompassing the C1 basin and three surrounding seamounts (i.e., NA, NLG, and MP4) with varying sampling locations in the bottom, hillside, and summit areas. These

samples represent three adjacent seamount sediments in the Northwest Pacific on a mesoscale level. In addition, to compare the viral sequences from seamount sediments with those from other marine environments, several publicly available metagenomic dataset were also included in this study, including Global Oceans Viromes 2 (GOV 2.0) database (https://doi.org/10.1016/j.cell.2019.03.040), cold seep database (https://doi.org/10.6084/m9.figshare.12922229) and trench database (https://doi.org/10.1038/s41396-021-00994-y).

Sampling strategy	Deep-sea seamount sediment samples were collected from the seamount region in the Northwest Pacific during the Dayang Crusie using Multi-tube sampler. No statistical methods were used to predetermine sample size. Seven sediment samples were chosen because they represent the mesoscale seamount region, which allows us to investigate the effects of the geographic features of seamounts on the microdiversity and biogeography of viruses on a mesoscale level.
Data collection	The V3-V4 region of the 16S rRNA gene was sequenced on the Illumina MiSeq platform by Majorbio Bio-Pharm Technology Co., Ltd. (Shanghai, China). The bulk metagenomic library was sequenced on an Illumina NovaSeq 6000 platform by Majorbio Bio-Pharm Technology Co., Ltd. (Shanghai, China). The virome library was sequenced on the Illumina HiSeq 2000 platform by Majorbio Bio-Pharm Technology Co., Ltd. (Shanghai, China). The publicly available metagenomic datasets were collected by co-authors Meishun Yu and Min Jin.
Timing and spatial scale	The seamount sediment samples were collected from NA, NLG, and MP4 seamounts in the Northwest Pacific in 2017. The publicly available metagenomic datasets were collected from 2022 to 2023.
Data exclusions	No data was excluded.
Reproducibility	All analyses were computational and it's straightforward to reproduce the findings according to the described methods.
Randomization	Randomization is not relevant since our study aims to discover the biogeography, community structure, genetic diversity, and ecological roles of viruses in seamount ecosystems. It is necessary to include all metagenomic datasets in this study.
Blinding	Blinding was not necessary for the development of this study as it is mostly descriptive and treats environmental sequencing samples that cannot be influenced by human manipulation.

Did the study involve field work? ☐ Yes ☒ No

Reporting for specific materials, systems and methods

We require information from authors about some types of materials, experimental systems and methods used in many studies. Here, indicate whether each material, system or method listed is relevant to your study. If you are not sure if a list item applies to your research, read the appropriate section before selecting a response.

Materials & experimental systems

n/a	Involved in the study	
☒	☐	Antibodies
☒	☐	Eukaryotic cell lines
☒	☐	Palaeontology and archaeology
☒	☐	Animals and other organisms
☒	☐	Clinical data
☒	☐	Dual use research of concern
☒	☐	Plants

Methods

n/a	Involved in the study	
☒	☐	ChIP-seq
☒	☐	Flow cytometry
☒	☐	MRI-based neuroimaging

Plants

Seed stocks	*Report on the source of all seed stocks or other plant material used. If applicable, state the seed stock centre and catalogue number. If plant specimens were collected from the field, describe the collection location, date and sampling procedures.*
Novel plant genotypes	*Describe the methods by which all novel plant genotypes were produced. This includes those generated by transgenic approaches, gene editing, chemical/radiation-based mutagenesis and hybridization. For transgenic lines, describe the transformation method, the number of independent lines analyzed and the generation upon which experiments were performed. For gene-edited lines, describe the editor used, the endogenous sequence targeted for editing, the targeting guide RNA sequence (if applicable) and how the editor was applied.*
Authentication	*Describe any authentication procedures for each seed stock used or novel genotype generated. Describe any experiments used to assess the effect of a mutation and, where applicable, how potential secondary effects (e.g. second site T-DNA insertions, mosiacism, off-target gene editing) were examined.*
